# Motilin and its receptor are expressed in the dorsal horn in a rat model of acute incisional pain: Intrathecal motilin injection alleviates pain behaviors

**DOI:** 10.3389/fnins.2023.1104862

**Published:** 2023-02-02

**Authors:** Yu Zhang, Jun Zhao, Nan Hu, Jing Wang, Xi Chen, Kaiyuan Wang, Yiqing Yin

**Affiliations:** ^1^Key Laboratory of Cancer Prevention and Therapy, Department of Anesthesiology, Tianjin Medical University Cancer Institute and Hospital, National Clinical Research Center for Cancer, Tianjin’s Clinical Research Center for Cancer, Tianjin, China; ^2^Department of Anesthesiology, Tianjin First Central Hospital, Nankai University, Tianjin, China

**Keywords:** motilin, motilin receptors, acute pain, spinal cord, animal model

## Abstract

**Aims:**

To observe the effects of intrathecal administration of motilin on pain behavior and expression of motilin (MTL)/motilin receptor (MTLR) in the spinal cord of a rat model of acute incisional pain.

**Methods:**

An incisional pain model was established in rats using a unilateral plantar incision. The rats were also injected intrathecally with 1, 5, or 25 μg of motilin. The mechanical withdrawal threshold (MWT) and thermal withdrawal latency (TWL) were determined. MTL/MTLR expression in the spinal cord was detected by western blotting and immunofluorescence. The expression of MTL in the spinal cord, stomach, duodenum, and plasma was determined by enzyme-linked immunosorbent assay (ELISA).

**Results:**

Motilin/motilin receptor were detected in the spinal cord. Spinal cord MTL/MTLR expression peaks at 2 h after modeling (*P* < 0.05) and start to decrease at 24 h (*P* < 0.05) to almost reach baseline levels at 72 h. The changes in gastric, duodenal, plasma, and spinal cord motilin levels correlated with MWT and TWL (all *R*^2^ > 0.82). The intrathecal injection of 1, 5, or 25 μg of motilin could increase the pain threshold of rats with incisional pain within 72 h in a dose-dependent manner.

**Conclusion:**

This study showed for the first time that MTL/MTLR are expressed in rats’ spinal dorsal horn. Acute pain increased MTL/MTLR expression in the spinal dorsal horn. Also, for the first time, this study showed that motilin intrathecal injection alleviates pain in rat models of acute incisional pain. These results suggest that MTL/MTLR could be a novel target for the management of acute pain.

## Introduction

Pain is probably the most common symptom and complaint in medicine (Raffaeli and Arnaudo, 2017; Chen et al., 2022). Acute pain can be due to any mechanical, thermal, or chemical injury to any part of the body (Basbaum et al., 2009; Chen et al., 2022). The stimulus will activate nociceptive receptors that will transmit the pain signal to the central nervous system (Chen et al., 2022). The management of acute pain usually involves drugs that can be classified as opioid and non-opioid analgesic agents (Zollner and Stein, 2007; Attal, 2019; Finnerup, 2019; Ghlichloo and Gerriets, 2022). Opioids are considered the most effective drugs for acute pain management (e.g., postoperative analgesia) (Zollner and Stein, 2007), but they are associated with serious drawbacks (addiction, tolerance, and side effects) that limit their use (Smith, 2012). Guidelines recommend the use of the minimal effective dose and for the shortest duration (Guy et al., 2017; Aubrun et al., 2019; Chakote and Guggenheimer, 2019; Cohen et al., 2022). Non-opioid agents are indicated for mild-to-moderate pain; there are several classes that can be used in specific situations (Finnerup et al., 2015; Attal, 2019; Finnerup, 2019; Ghlichloo and Gerriets, 2022), but they are generally less effective than opioids. Therefore, there is a need to investigate effective analgesia methods with minimal drawbacks.

Motilin (MTL) is a polypeptide composed of 22 amino acids secreted by intestinal chromaffin cells. It acts on gastrointestinal smooth muscle and intestinal nervous system, playing a role in gastrointestinal motility (Kitazawa and Kaiya, 2021; Tack et al., 2021), and is involved in various gastrointestinal diseases (Van den Houte et al., 2020; Wang T. et al., 2020; Ko et al., 2021; Zhang et al., 2021). MTL binds to MTL receptors (MTLRs) that are located primarily in the enteric nerves, smooth muscle, and gastric vagal nerve terminals of the gastrointestinal (GI) tract (Kitazawa and Kaiya, 2021; Li et al., 2021; Miedzybrodzka et al., 2021). MTL is involved in regulating gastric motility through the vagus nerve (Sanger et al., 2009; Liu et al., 2010; Javid et al., 2013). The vagal innervation of gastric motility depends mainly on the excitability of the premotor neurons located in the dorsal nucleus of the vagus nerve in the stomach (Liu et al., 2010; Javid et al., 2013).

Motilin and motilin receptors are also found in the central nervous system (hippocampus, hypothalamus, amygdala, etc.), where they participate in the gut-brain axis (Zhao et al., 2014, 2018; Wang C. et al., 2020). In addition to participating in gastrointestinal motility, MTL participates in the regulation of feeding and emotions (Simpson et al., 2021; Tack et al., 2021). MTL increase in patients taking certain types of antidepressants, suggesting an indirect association between changes in MTL and the response to these drugs. In addition, MTL could be correlated with processes or events opposed to depression (Rajkumar, 2021). The antidepressant mirtazapine (Jiang et al., 2016) and certain phytochemicals (Du et al., 2014; Ko et al., 2021) improve depressive and gastrointestinal symptoms in patients with functional dyspepsia (FD), and MTL levels are also increased. MTL and the MTL agonist erythromycin increase GABAergic transmission, reducing anxiety-like responses to stress (Feng et al., 2013).

Acute incisional pain can participate in the regulation of MTL expression in the hippocampus, plasma, gastric body, and duodenum (Zhao et al., 2014). Intrathecal administration of appropriate doses of opioids combined with low-dose naloxone analgesia gradually restores MTL close to the physiological levels, suggesting a link between pain and MTL expression (Zhao et al., 2014). MTL and MTLRs play an important role in antidepressant treatment (Ko et al., 2021; Rajkumar, 2021; Zhou and Wang, 2021), and as is well-known, pain is closely related to depression (Reckziegel et al., 2016; Albrecht et al., 2021).

The spinal cord is an important transmission center of pain signals (Zhang et al., 2017; Chen and Sun, 2020; Tam and Salter, 2021). The spinal dorsal horn is the first synapse in the pain pathway and is a key target for controlling spinal nociceptive transmission (Lu et al., 2018). The descending regulation of spinal nociceptive processing originates from many brain regions, and the brainstem plays a critical role in the experience of pain (Heinricher et al., 2009). It is unclear whether MTL and MTLR coexist at the spinal cord level, how they are distributed, and how they change with acute pain.

Therefore, to clarify the dynamic relationship between spinal MTL/MTLRs and acute pain, verify whether MTL has analgesic or pain-inducing effect, and preliminarily explore its possible mechanism, the present study investigated the specific distribution of MTL and MTLRs at the spinal cord level, changes of MTL/MTLRs expression in response to different degrees of pain, and the effects of intrathecal injection of different doses of MTL on pain behavior. The findings could provide a basis for clinical research and the development of new drugs for gastrointestinal motility, emotion regulation, and pain. This study is the first to examine the involvement of central nervous system MTL/MTLR in pain.

## Materials and methods

### Animals

Adult Sprague–Dawley rats (*n* = 135, 6–8 weeks, 180–220 g) were obtained from the Experimental Animal Center of Tianjin Medical University Cancer Institute and Hospital (Tianjin, China) and Beijing Vital River Laboratory Animal Technology Co., Ltd., and housed under controlled relative humidity (20–30%), temperature (23 ± 2°C), and 12/12 h light/dark cycle (light from 08:00 to 20:00) with access to food and water *ad libitum*. Before experimental procedures, the animals were allowed 7 days of acclimation, and efforts were made to limit any distress. The protocol was approved by the Animal Care and Use Committee of the Animal Ethical and Welfare Committee of Tianjin Medical University Cancer Institute and Hospital (approval #NSFC-AE-2020210). The study was conducted in accordance with the Declaration of the National Institutes of Health’s Guide for the Care and Use of Laboratory Animal (Publication No. 80-23, revised 1996) and the policies on the use of laboratory animals issued by the International Association for the Study of Pain (IASP).

### Study reagents

The MTL enzyme-linked immunosorbent assay (ELISA) kit was purchased from Gene Biotech Company (Shanghai, China). The doses of each drug were determined based on preliminary experiments. The MTL (NBP2-93868; Novus Biologicals, Littleton, CO, USA), MTLR (DF4993; Affinity BIO, Scoresby, Australia), IgG-FITC (Santa Cruz Biotechnology, Santa Cruz, CA, USA), IgG-CY3 (Santa Cruz Biotechnology, Santa Cruz, CA, USA), IgG-CY5 (Santa Cruz Biotechnology, Santa Cruz, CA, USA), and HRP-conjugated (GB23303; Servicebio, Woburn, MA, USA) antibodies were purchased.

### Animal grouping and experimental protocol

Seventy-eight rats were randomly divided into two groups: the control group (group C, *n* = 6) and the incisional pain group (group P, *n* = 72). Group P underwent incisional pain modeling. Six rats in the two groups were randomly selected. The mechanical withdrawal threshold (MWT) and thermal withdrawal latency (TWL) were measured 24 h before operation (T0), 2 h after operation (T1), 24 h after operation (T2), 48 h after operation (T3), and 72 h after operation (T4).

In order to demonstrate the effect of MTL on acute pain, we performed direct intrathecal injections of different doses of MTL in rats. Forty-five healthy male SD rats (6–8 weeks, 180–220 g) were randomized into five groups (*n* = 9/group): control group (group C), incisional pain group (group P), incisional pain + 1 μg MTL group (group 1MP), incisional pain + 5 μg MTL group (group 5MP), and incisional pain + 25 μg MTL group (group 25MP). The MWT and TWL were measured at T0, T1, T2, T3, and T4.

To measure locomotor function, we performed open field testing in which 12 SD rats (6–8 weeks, 180–220 g) were randomized into two groups: saline group (group Vehicle, *n* = 6) and intrathecal MTL group (group MTL, *n* = 6). 25 μl of normal saline was injected intrathecally in group Vehicle and 25 μg of MTL in 25 μl saline was injected in group MTL. Locomotor behavior was recorded 2 h after injection.

### Intrathecal catheterization

A lumbar intrathecal catheter was inserted using a modified version of a reported technique for subarachnoid drug administration (Storkson et al., 1996). Briefly, rats were anesthetized with 4% chloral hydrate (400 mg/kg, intraperitoneal injection). The lumbar skin was shaved, cleaned, and incised. The intervertebral space between L5 and L6 was punctured with a hypodermic needle, and a PE10 tubing (outer diameter of 0.55 mm, inner diameter of 0.30 mm; AniLab Software and Instruments Company, China) was inserted. The catheter localization was verified by injecting 10 μl of 2% lidocaine: only the rats with a brief bilateral hind limb paresis after injection (indicated catheterization success) were used for the incisional pain model and intrathecal MTL injection experiments (Ray et al., 2011; Hou et al., 2016). The experiments started 7 days after successful catheter placement. At 20 min before modeling, 25 μl of normal saline was injected intrathecally in groups C and P, while the 1MP, 5MP, and 25MP were injected with the proper dose of MTL in 25 μl of normal saline. After 20 min, the right plantaris muscle incision was made in the pain groups.

### Plantar incision acute pain model

The rat hind paw plantar incisional pain model was established as previously described (Brennan et al., 1996). After the rats were anesthetized using sevoflurane, a 1 cm longitudinal incision was made through the skin and fascia of the plantar aspect of the right hind paw. The underlying plantar muscle was elevated and incised longitudinally, leaving the muscle origin and insertion intact. The skin was apposed with two mattress sutures using 5-0 nylon threads. After surgery, the animals were allowed to recover in their cages.

### Mechanical withdraw threshold

The MWT was determined as previously described (Hohmann et al., 2017). In order to determine the MWT of the rats, a dynamic plantar esthesiometer (Ugo Basile, Comerio, Italy) was used. The rats were put on an elevated grid in test cages at least 1 h before the measurement to allow accommodation. Then, baseline measurements were performed before incisional pain modeling. A steel filament was pushed against the plantar side of the hind paw with a linear ascending force (0–5 g over 10 s, in 0.5 g/s intervals) until a fast withdrawal of the paw was observed. The time between the first contact of the steel rod and the paw withdrawal was measured in seconds (paw withdrawal latency, PWL), and a cut-off time of 20 s was set.

### Thermal withdrawal latency

The TWL was determined as previously described (Cao et al., 2015). TWL was evaluated using the Plantar Test apparatus (UgoBasile, Comerio, Italy). The Plantar Test apparatus was calibrated before TWL measurement. Each animal was allowed to habituate to the environment for 1 h before any measurements. A mobile heat source with an infrared intensity of 70 W was placed under the hind paw. TWL was defined as the duration from starting heat to inducing the paw to withdraw. Each foot was measured three times, and then its average was calculated. There was a 5-min rest period between measurements.

### ELISA

Motilin levels in the plasma, stomach, duodenum, and spinal cord were determined using rabbit-specific ELISA kits per the manufacturer’s instructions. Six rats in each group were sacrificed by spine dislocation before incision pain at T0–T4. The plasma, stomach, duodenum, and spinal cord samples were quickly prepared for MTL assessment.

### Western blotting

The spinal cord samples from six rats at each time point were quickly extracted and stored in liquid nitrogen. Tissue samples were homogenized at pH 7.4 in lysis buffer containing Tris (20.0 mM), sucrose (250.0 mM), sodium orthovanadate (0.03 mM), magnesium chloride (2.0 mM), ethylenediaminetetraacetic acid (2.0 mM), ethylene glycol tetraacetic acid (2.0 mM), phenylmethylsulfonyl fluoride (2.0 mM), dithiothreitol (1.0 mM), and a protease inhibitor cocktail (0.02% v/v; G2006, Servicebio, Wuhan, China). The homogenates were centrifuged at 5,000 × *g* for 30 min at 4°C. The supernatants were stored at –80°C. Protein concentration was measured using the Bradford method. Proteins (30 μg) were separated by 10% SDS-PAGE and transferred onto a nitrocellulose membrane (G6014, Servicebio, Wuhan, China). The membranes were incubated overnight at 4°C with primary polyclonal rabbit anti-human MTLR IgG (1:1,000; DF4993; Affinity biosciences, USA) and Motilin Antibody (1:1,000; NBP2-93868; Novus Biologicals, Littleton, CO, USA). After washing with Tris-buffered saline Tween 20 (TBST), the membrane was incubated for 1 h with secondary antibody conjugated with alkaline phosphatase (1:500) at room temperature. The immune complexes were detected using a nitro blue tetrazolium/5-bromo-4-chloro-3-indolyl phosphate assay kit (Sigma, St. Louis, MO, USA). Western blot densitometry analysis of signal intensity was performed using Adobe Photoshop software (Adobe, San Jose, CA, USA). The blot density from control groups was set at 100%.

### Immunofluorescence

At T0, T1, and T2, the rats were anesthetized with 10% chloral hydrate (300 mg/kg, i.p.) and perfused intracardially with 200 ml of saline followed by 500 ml of ice-cold 4% paraformaldehyde in 0.2 M phosphate buffer (pH 7.4). The lumbar spinal cord (at L4-6) was removed immediately, postfixed in the same fixative overnight, and subsequently transferred to 30% sucrose (w/v) in phosphate buffer and stored at 4°C for several days. The specimens were then embedded with OCT at –20°C, and 30-μm transverse series sections were cut on a cryostat (CM1900, Leica, Wetzlar, Germany) and collected in 0.01 M phosphate-buffered saline (PBS, pH 7.4).

The sections were immersed in antigen retrieval buffer, heated in a microwave oven for antigen retrieval at a subboiling temperature, cooled naturally, and washed with PBS three times, 3 min/time. The sections were blocked with 3% BSA at room temperature for 30 min. The sections were incubated with a rabbit anti-MTL antibody (1:1,000) and rabbit anti-MTLR (1:200) overnight at 4°C, followed by a secondary antibody conjugated with HRP and CY3-TSA (1:500, servicebio) or a goat anti-rabbit secondary antibody conjugated with 488 (1:400). In order to identify the expression of MTLR on neurons of the dorsal horn, double immunofluorescence staining for both MTLR and Tubb3 was performed simultaneously with rabbit anti-MTLR antibody (1:200) and mouse anti-Tubb3 antibody (1:100) overnight at 4°C. The sections were rinsed in PBS and incubated in goat anti-rabbit IgG coupled to if488 (1:400) and Goat anti-mouse IgG coupled to Cy5 (1:400) in the dark for 50 min at room temperature. The nuclei were counterstained with DAPI. After washing with PBS, all the sections were mounted on gelatin-coated slides and coverslipped with glycerol. Images were captured using a laser-scanning confocal microscope (TCS SP2; Leica, Wetzlar, Germany). Image-Pro Plus 6.0 (Media Cybernetics, Inc., Rockville, MD, USA) was used for analysis.

### Open field testing

To measure locomotor function, we performed open field testing in which rats were placed in the center of a 100 × 100 cm chamber and locomotor activity was recorded by an overhead webcam connected to a laptop computer, and animals’ movements were automatically tracked for 30 min using Visualtrack. The total distance traveled and mean speed during the 30-min period were analyzed.

### Data analysis

The data were expressed as mean ± SEM. Statistical analyses were performed using the unpaired Student’s *t*-test or one- or two-way ANOVA in behavioral experiments (ANOVA with repeated measures). All statistical analyses were performed using GraphPad Prism 9 (GraphPad Software Inc., San Diego, CA, USA). Pearson correlation analysis was performed between the MTL levels and MWT or TWL at each time point. Two-sided *P*-values < 0.05 were considered statistically significant.

## Results

### Behavioral changes in rats with acute incisional pain

**FIGURE 1:**
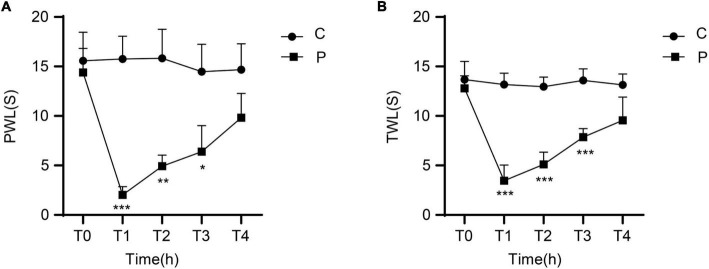
Changes in mechanical withdrawal threshold (MWT) measured as paw withdrawal latency (PWL) and thermal withdrawal latency (TWL) of rats at different time points after incisional pain modeling (group P). *n* = 6 rats/group/time point. **(A)** MWT. **(B)** TWL. *P < 0.05, **P < 0.01, and ***P < 0.001, P group vs. C group.

There were no significant differences in MWT between the C and P groups at T0. Compared with the C group, the animals in the P group showed significantly decreased MWT at T1, T2, and T3, especially at T1. MWT gradually increased from T2 and returned close to baseline levels at T4 (Figure 1A). There were no significant differences in TWL between the C and P groups at T0. Compared with the C group, the P group showed significantly decreased TWL at T1, T2, and T3, especially at T1. TWL gradually increased from T2 and returned close to the baseline levels at T4 (Figure 1B). The results suggest that in the P group, the severest pain was observed at T1, which was alleviated at T4.

### Spinal cord immunofluorescence of MTL and MTLR

**FIGURE 2:**
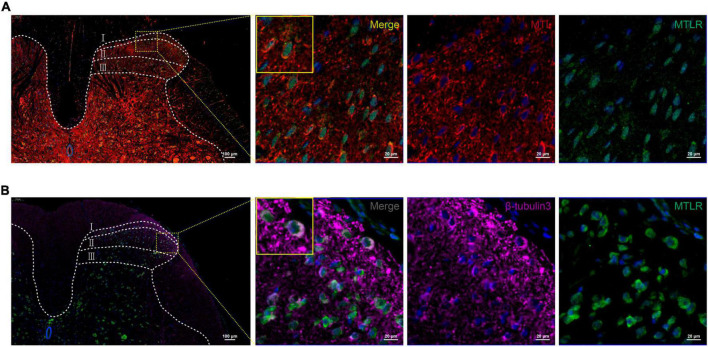
**(A)** Motilin (MTL) and MTL receptor (MTLR) fluorescence co-labeling in the spinal dorsal horn 2 h after acute incisional pain. **(B)** Co-labeling of MTLR and β-tubulin III fluorescence in the spinal dorsal horn. MTL (red), MTLR (green), β-tubulin III (violet), and DAPI (blue). Scale bar = 100 μm/20 μm.

**FIGURE 3:**
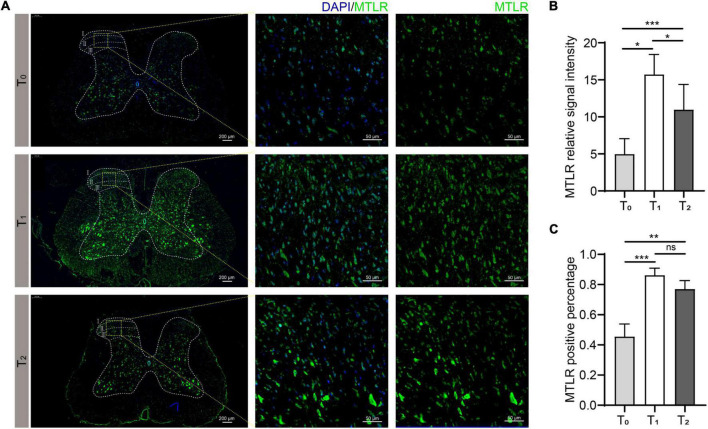
Motilin receptor (MTLR) fluorescence in the spinal dorsal horn before surgery (T0), 2 h after acute incisional pain (T1), and 24 h after surgery (T2). **(A)** Immunofluorescence images. Scale bar = 200 μm/50 μm. **(B)** MTLR relative signal intensity. **(C)** MTLR positive percentage. *n* = 6 rats/group/time point. ns, not significant; *P < 0.05, **P < 0.01, and ***P < 0.001.

In order to verify whether MTL and MTLR are found in the spinal cord and what is their specific distribution, the changes in MTL and MTLR were examined by immunofluorescence at T0, T1, and T2 in the P group. MTL (red), MTLR (green), and neuronal markers were found in the superficial layer of the dorsal horn in the spinal cord. MTL and MTLR, as well as β-tubulin III and MTLR, were co-labeled in the superficial layers of the dorsal horn (Figure 2). The evidence suggests that MTL and MTLR interact in the cells located in the superficial layers of dorsal horn in the state of acute pain and provides a histological basis for possibilities that MTL and MTLR may play biological roles in the specific sensory regulation region. However, the current results only managed to indicate the existence of MTLR on dorsal horn neurons. The results also showed that with the decrease in pain threshold, the expression of MTLR in the superficial layers of the dorsal horn was upregulated. When the pain was most obvious at T1, the expression of MTLR was significantly higher than at T0, whereafter, the expression was downregulated at T2 but still higher than at T0 (Figure 3).

### Dynamic expression of MTL in gastric body, duodenum, plasma, and spinal cord

**FIGURE 4:**
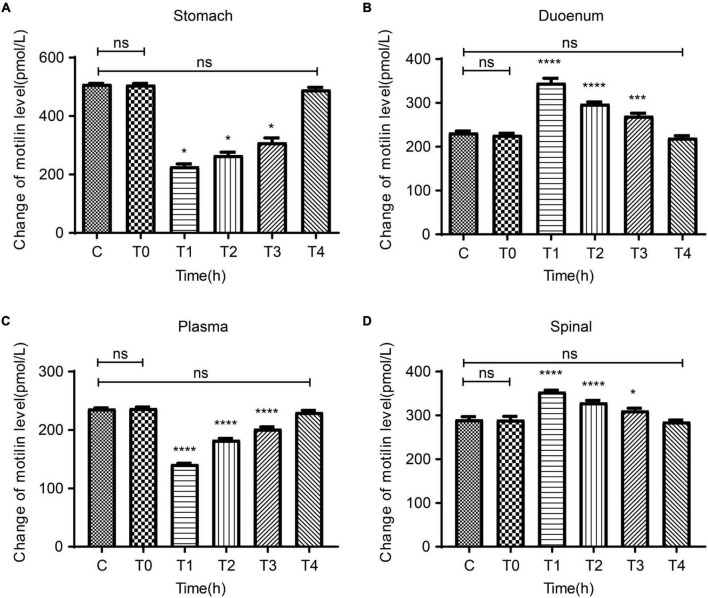
Dynamic changes in motilin (MTL) levels before surgery (T0) and at 2 h (T1), 24 h (T2), 48 h (T3), and 72 h (T4) after surgery in the acute incisional pain model (P group) and controls (C group). **(A)** Stomach. **(B)** Duodenum. **(C)** Plasma. **(D)** Spine cord. *n* = 6 rats/group/time point. ns, not significant; *P < 0.05, ***P < 0.001, and ****P < 0.0001.

In order to observe the relation between the degree of pain and the central and peripheral gastric dynamic expression levels, we collected the stomach, duodenum, plasma, and spinal cord of rats in the C and P groups at T0-T4 to test MTL levels. There were no differences between groups C and P regarding the MTL levels in the stomach, duodenum, plasma, and spinal cord at T0. Compared with T0, the MTL levels in the gastric body of group P decreased at T1 and gradually increased at T2–T4 (Figure 4A). Compared with T0, the duodenal MTL levels were increased at T1 and gradually decreased at T2–T4 (Figure 4B). Compared with T0, the plasma MTL levels were decreased at T1, began to increase at T2, and gradually increased at T2–T4 (Figure 4C). In the spinal cord, compared with group C, MTL levels were increased at T1 and gradually decreased at T2–T4 (Figure 4D). Therefore, the expression of MTL varies in time after pain stimulation and varies according to organs/tissues. In the spinal cord, MTL varied with time after pain stimulation.

### Correlation between pain threshold and MTL expression in rats with incisional pain

**FIGURE 5:**
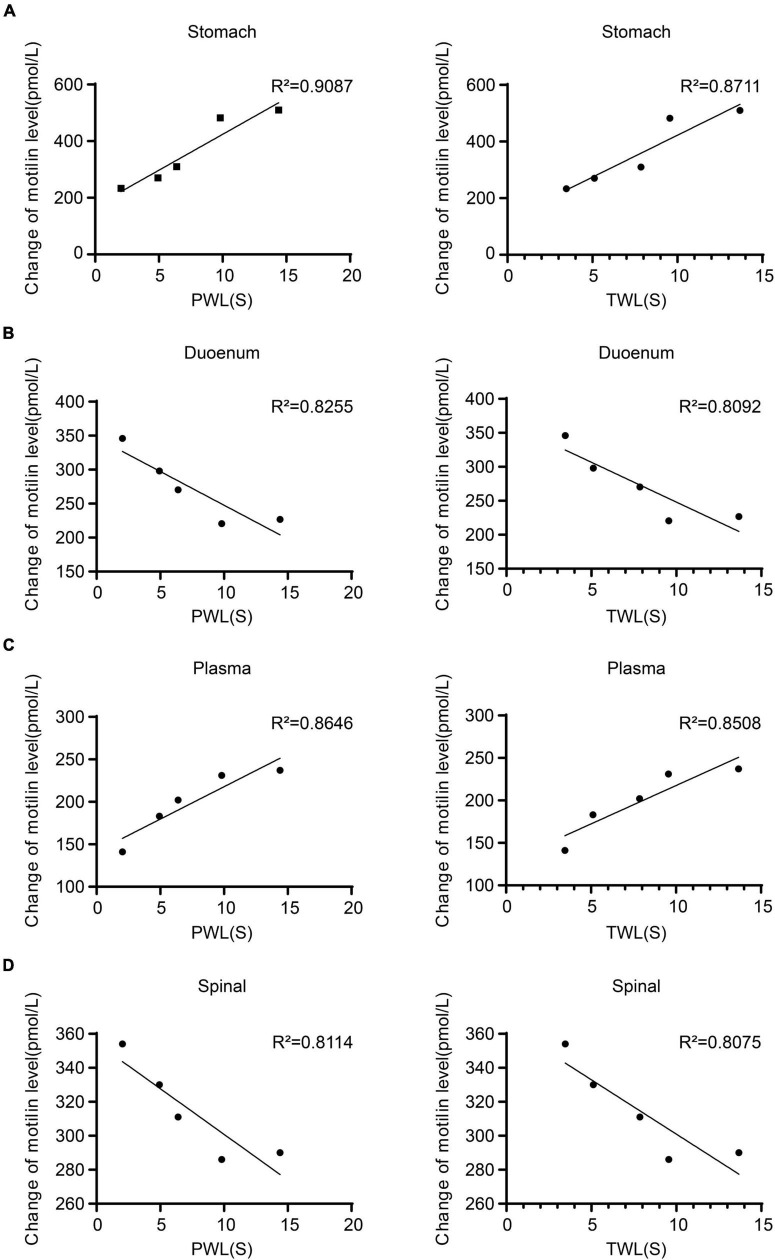
Regression curves of the correlation between mechanical withdrawal threshold (MWT) measured as paw withdrawal latency (PWL) and thermal withdrawal latency (TWL) and motilin (MTL) expression in **(A)** gastric body, **(B)** duodenum, **(C)** plasma, and **(D)** spinal cord in rats with incisional pain.

The expression of MTL was positively correlated with MWT and TWL in the gastric body (MWT: *R*^2^ = 0.9087; TWL: *R*^2^ = 0.8711), plasma (MWT: *R*^2^ = 0.8255; TWL: *R*^2^ = 0.8092). The expression of MTL in the duodenum (MWT: *R*^2^ = 0.8646; TWL: *R*^2^ = 0.8508) and spinal cord (MWT: *R*^2^ = 0.8114; TWL: *R*^2^ = 0.8075) were negatively correlated with MWT and TWL (Figure 5). Therefore, MTL expression is correlated with the changes of pain thresholds.

### Expression of MTL and MTLR in the spinal cord by western blotting

**FIGURE 6:**
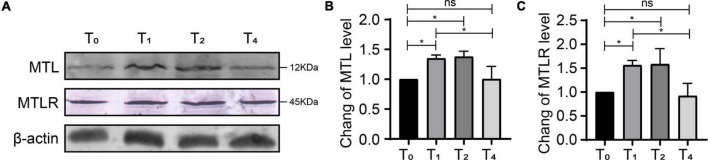
Dynamic changes of motilin (MTL) and MTL receptor (MTLR) in the dorsal horn in rats with acute incisional pain. **(A)** Western blot. **(B)** MTLR. **(C)** MTL. *n* = 6 rats/group/time point. ns, not significant; *P < 0.05. P0, 24 h before surgery; P1, 2 h after surgery; P2, 24 h after surgery; and P4, 72 h after surgery.

Compared with T0, the expression of MTL and MTLR in group P was increased significantly at T1 and T2, corresponding to the time points of ongoing acute pain. At T4, the behavioral pain indexes tended toward baseline levels, and the expression of MTL and MTLR gradually decreased and were not significantly different from baseline (Figure 6). Therefore, the expression of MTL and MTLR is a dynamic process in response to acute pain.

### Effect of MTL infusion on pain

**FIGURE 7:**
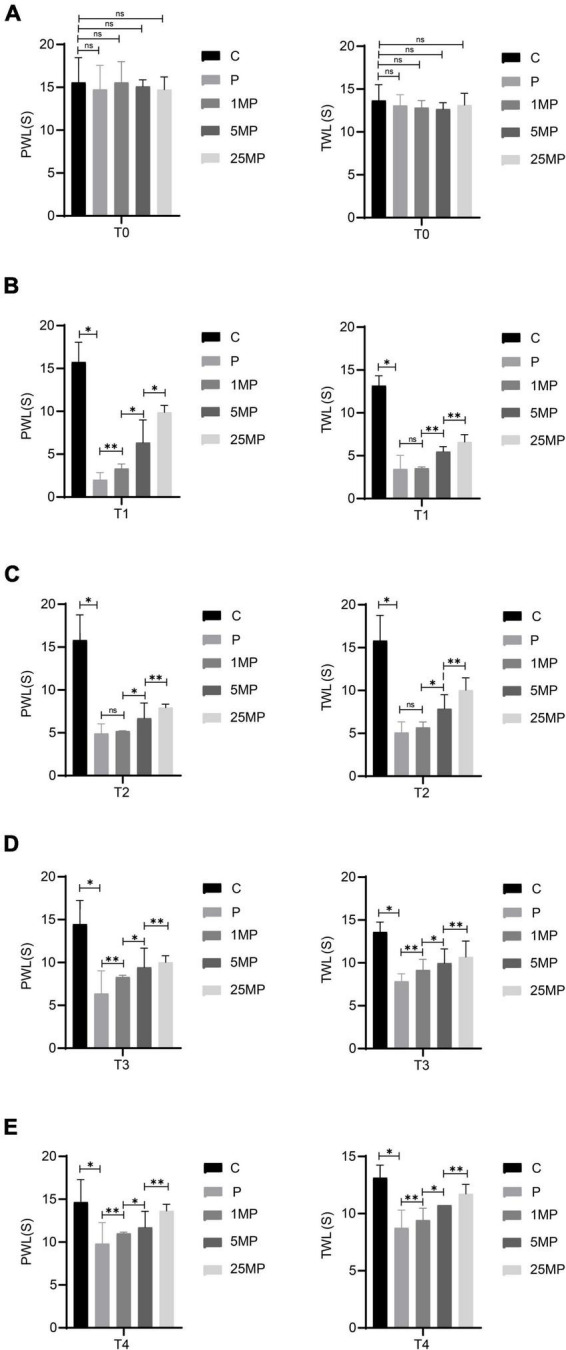
Effect of intrathecal injection of motilin (MTL) on the mechanical withdrawal threshold (MWT) measured as paw withdrawal latency (PWL) and thermal foot withdrawal response latency (TWL) in the control (C) and pain model groups (P) at 24 h before operation [T0; **(A)**], 2 h after the operation [T1; **(B)**], 24 h after the operation [T2; **(C)**], 48 h after the operation [T3; **(D)**], and 72 h after the operation [T4; **(E)**]. 1MP, pain with 1 μg of MTL; 5MP, pain with 5 μg of MTL; and 25MP, pain with 25 μg of MTL. ns, not significant; *P < 0.05, **P < 0.01.

The behavioral results (Figure 7) showed that intrathecal injection of 1, 5, or 25 μg of MTL could increase the pain threshold of rats with incisional pain at T4, suggesting that MTL had a definite and long-lasting analgesic dose-dependent effect. It is unclear whether there is a ceiling effect to this dose-dependence for there were currently no experimental group with a higher dose of MTL.

### Locomotor function evaluation in rats with MTL intrathecal injection

**FIGURE 8:**
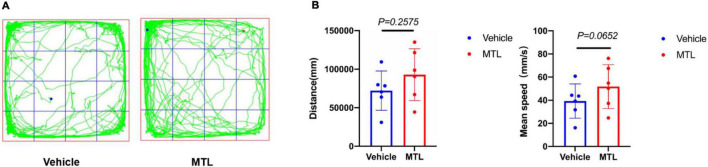
**(A)** Trajectory chart of rats with intrathecal injection of 25 μl of normal saline (group Vehicle) and 25 μg of MTL (group MTL) during 30-min period. **(B)** Distance (mm) and mean speed (mm/s) of rats during 30-min duration in the control (Vehicle) and MTL group (MTL) in the open field testing 2 h after injection. *n* = 6 rats/group. Group Vehicle vs. group MTL, *P* < 0.05, significant.

The behavioral results (Figure 8) showed that the total distance traveled and mean velocity of rats with intrathecal injection of 25 μg of MTL did not differ significantly from the rats with the injection of normal saline during the 30-min period of the open field testing at 2 h after injection. The findings provide evidence that 25 μg of MTL intrathecal injection does not compromise locomotor function of rats.

## Discussion

This study aimed to observe the effects of intrathecal administration of MTL on pain behavior and expression of MTL and MTLR in the spinal cord of a rat model of acute incisional pain. This study showed for the first time that MTL/MTLR are expressed in rats’ spinal dorsal horn. Acute pain increased MTL/MTLR expression in the spinal dorsal horn. Also, for the first time, this study showed that MTL intrathecal injection alleviated pain in rat models of acute incisional pain. The findings presented here provide a new basis that MTL might be involved in regulating peripheral analgesia at the spinal cord level, suggesting an analgesic mechanism for an MTL and MTLR signaling pathway. MTL and MTLR could be used as potential novel analgesic methods in managing acute pain.

Injurious stimuli induce an inflammatory response that produces acute pain through the action of neural reflexes and inflammatory factors, with an increase in sympathetic excitability and significant suppression of vagal excitability (Bonaz et al., 2016), resulting in a decrease in gastric somatic gastrin levels and a significant decrease in gastric motility (Guo et al., 2011; Kitazawa and Kaiya, 2021). The non-adrenergic non-cholinergic (NANC) nerves are believed to provide the main inhibitory innervation of the gut, and these neurons are known to release nitric oxide and vasoactive intestinal polypeptides (Zhao et al., 2014). Thus, acute pain can mediate the downregulation of MTL expression through a combination of the sympathetic-vagal pathway and NANC neural pathway, which leads to decreased gastric motility. These results support the decreased gastric and plasma levels of MTL observed after surgery. On the other hand, duodenal MTL expression increased after modeling, which is consistent with a study that showed that the mechanisms of MTL-induced contractions differed among various regions of the GI tract of quail (Apu et al., 2016).

In the present study, MTL and MTLR were detected in the spinal cord of rats and increased after pain modeling, which has not been reported before. It suggests that MTL in the hippocampus and spinal cord can be in a common state of increased responsiveness under acute incisional pain stimulation, but whether the conduction mechanism is the same signal pathway has not been reported. MTL acts as a stimulatory factor in the hippocampus to regulate gastric motility, perhaps through the nitric oxide pathway (Xu et al., 2008). However, the mechanism of pain regulating spinal MTL is still unclear. Nevertheless, these results provide a preliminary morphological basis to establish the hypothesis that MTL and its receptor play a biological role in the spinal cord and pain. The results showed that MTL and MTLR were largely co-labeled in the superficial layers of the dorsal horn, which suggested the morphological interaction of MTL and MTLR in the superficial cells (cell type not determined). Furthermore, Co-labeling of MTLR and β-tubulin III strongly confirmed the expression of MTLR in the neurons of the superficial layers of the dorsal horn where the nociceptive stimuli are processed and relayed. Compared with the baseline, the immunofluorescence expression of MTLR was significantly upregulated at 2 and 24 h, however, the expression began to decrease at 2 h after the operation, and when it reached 24 h, MTL level was still higher than baseline. Western blot further supplemented the results by showing similar upregulation of MTL and MTLR expression in response to pain at 2 and 24 h, following by a decrease at 72 h. We also found that the intrathecal injection of 1, 5, and 25 μg of MTL could improve the MWT and TWL within 72 h after the operation with no obviously adverse effect on the locomotor function at 25 μg of MTL injection. Moreover, the degree of analgesia increased in a dose-dependent manner.

Therefore, this study demonstrated for the first time the analgesic effect of an intrathecal administration of MTL and suggested MTL as a possible endogenous analgesic substance. It partly explains why the expression trend of MTL and its receptors at central and peripheral levels changed with the degree of pain after the pain stimuli emerged. It has been confirmed that many endogenous peptide analgesics (e.g., OT, orexin A and B, opioid peptides, and serotonin) achieve sensory regulation at the presynaptic or postsynaptic levels in the excitatory or inhibitory pathways of the dorsal horn (Becquet et al., 2019), but their regulatory mechanisms are different. Both Orexin A and B can cause membrane depolarization of dorsal neurons and enhance GABAergic inhibitory signaling (Becquet et al., 2019). Under peripheral nociceptive stimulation, OT and OTR showed high expression in the corresponding spinal cord segments; more interestingly, injury also resulted in a 6.5-fold increase in OTR mRNA expression in the ipsilateral L4 dorsal root ganglion (Jiang et al., 2016). OT signal transduction in the spinal cord is sustained in recovering mechanical pain sensitivity after surgical nerve injury (Jiang et al., 2016). To some extent, it suggests that nociceptive stimulation can lead to increased expression of endogenous analgesic substances at the level of the spinal cord, which is a typical example of the body’s self-protection and activation of endogenous analgesia. How MTL responds to pain and manages pain will have to be examined in future studies.

Of course, this study had limitations. The other transmitters and proteins involved in pain were not examined and may correlate with MTL and MTLR. The study didn’t manage to clarify the specific cell type in the dorsal horn in which MTL and MTLR interact or function, neither did the study examined the signaling pathways. Further study is needed to focus on these concerns. The MTL dose of 25 μg did not reach a plateau in pain response, and future studies should use higher doses.

## Conclusion

This study showed for the first time that MTL/MTLR are expressed in rats’ spinal dorsal horn. Acute pain increased MTL/MTLR expression in the spinal dorsal horn. Also, for the first time, this study showed that MTL intrathecal injection alleviates pain in rat models of acute incisional pain. Hence, MTL/MTLR could be a novel target for the management of acute pain.

## Data availability statement

The original contributions presented in this study are included in the article/supplementary material, further inquiries can be directed to the corresponding authors.

## Ethics statement

This animal study was reviewed and approved by the Animal Care and Use Committee of the Animal Ethical and Welfare Committee of Tianjin Medical University Cancer Institute and Hospital (approval #NSFC-AE-2020210).

## Author contributions

YY, YZ, JZ, and KW: study concept and design, funding, study supervision, and writing of the manuscript. YZ and JZ: acquisition of data. YZ, JZ, NH, JW, and XC: analysis and interpretation of data. All authors contributed to the article and approved the submitted version.
